# Erythropoietin Intensifies the Proapoptotic Activity of LFM-A13 in Cells and in a Mouse Model of Colorectal Cancer

**DOI:** 10.3390/ijms19041262

**Published:** 2018-04-23

**Authors:** Anna Tankiewicz-Kwedlo, Justyna Magdalena Hermanowicz, Krystyna Pawlak, Robert Czarnomysy, Krzysztof Bielawski, Izabela Prokop, Dariusz Pawlak

**Affiliations:** 1 Department of Monitored Pharmacotherapy, Medical University of Bialystok, Mickiewicza 2C, 15-222 Bialystok, Poland; krystynapawlak@poczta.onet.pl; 2 Department of Pharmacodynamics, Medical University of Bialystok, Mickiewicza 2C, 15-222 Bialystok, Poland; justyna.hermanowicz@umb.edu.pl (J.M.H.); dariuszpawlak@poczta.onet.pl (D.P.); 3 Department of Clinical Pharmacy, Medical University of Bialystok, Mickiewicza 2C, 15-222 Bialystok, Poland; 4 Department of Synthesis and Technology of Drugs, Medical University of Bialystok, Mickiewicza 2C, 15-222 Bialystok, Poland; robert.czarnomysy@umb.edu.pl (R.C.); kbiel@umb.edu.pl (K.B.); 5 Department of Medicinal Chemistry, Medical University of Bialystok, Mickiewicza 2C, 15-222 Bialystok, Poland; izabela.prokop@umb.edu.pl

**Keywords:** apoptosis, Bruton’s tyrosine kinase, colon cancer, erythropoietin, LFM-A13, xenografts

## Abstract

The Bruton’s tyrosine kinase (BTK) inhibitor LFM-A13 has been widely employed as an antileukemic agent, but applications in solid cancer have been found recently. The compound promotes apoptosis, has an antiproliferative effect, and increases cancer cell sensitivity to chemotherapy drugs. We decided to assess the impact of the simultaneous use of erythropoietin (Epo) and LFM-A13 on signal transduction in colon DLD-1 and HT-29 cells, as well as in tumor xenografts. The induction of apoptosis by Epo and LFM-A-13 in the cells was confirmed by phosphatidylserine externalization, loss of mitochondrial membrane potential, and modulation of the expression of apoptotic protein BAX and antiapoptotic protein BCL-2 in colon adenocarcinoma cells. Nude mice were inoculated with adenocarcinoma cells and treated with Epo and LFM-A13 in order to evaluate the degree of tumor regression. The simultaneous use of Epo and LFM-A13 severely inhibited cell growth, activated apoptosis, and also inhibited tumor growth in xenografts. The addition of Epo to LFM-A13 intensified the antiproliferative effect of LFM-A13, confirmed by the loss of mitochondrial membrane potential and the accumulation of apoptotic colon cancer cells with externalized phosphatidylserine (PS). These preclinical results suggest that the combination of Epo and LFM-A13 has a high proapoptotic activity and should be tested in the clinic for the treatment of solid tumors such as colon cancer.

## 1. Introduction

A dysfunctional apoptotic process can lead to both pathogenesis of colorectal cancer and its resistance to chemotherapy and radiotherapy [1]. Apoptosis, a cellular form of cell death, is currently an area interest for clinicians involved in colorectal cancer treatment and management [2].

Identifying novel intracellular proteins that play a crucial role in the growth and survival of colon cells has provided more effective therapies for cancer patients. Protein tyrosine kinase inhibitors are currently among the most promising drug types. Tyrosine kinases which often undergo mutations are the most commonly identified dominant oncogenes. Overexpression or activating mutation of these enzymes cause increased tumor cell proliferation and apoptosis evasion, while promoting angiogenesis and metastasis.

LFM-A13 is an active metabolite of leflunomide, designed using computer modeling techniques. It is the first inhibitor of Bruton’s tyrosine kinase (BTK), a cytoplasmic protein tyrosine kinase involved in signal transduction pathways regulating growth, maturation, differentiation, and cell viability. BTK is also an upstream activator of multiple antiapoptotic signaling molecules and networks, including the phosphatidylinositol-3-kinase/protein kinase B pathway [3]. Vassilev et al. demonstrated that BTK plays a role in the marked resistance of chicken B cell line DT40 as well as human leukemic B-cell precursors against Fas-mediated apoptosis. BTK links with Fas receptor via its kinase and pleckstrin homology (PH) domains and prevents the FAS–FADD (Fas-associated protein with death domain) interaction, which is essential for the recruitment and activation of FLICE (inhibitory proteins: regulators of death receptor-mediated apoptosis) by Fas during the apoptotic signal [4]. Recent data suggest the implication of BTK in signal transduction pathways affecting gene transcription [5,6]. Inhibition of BTK, specifically, by the irreversible inhibitor ibrutinib, leads to the upregulation of apoptosis-related genes [7]. Bruton tyrosine kinase (BTK) has been demonstrated to be a key element of the βcR signaling pathway. βcR crosslinking causes BTK to interact with the inner leaflet of the plasma membrane to constitute the βcR signalosome, which subsequently stimulates downstream pathways, including nuclear factor (NF)-κB. A strong positive correlation in gene expression between BCL-2 and BTK was also proved. In mantle cell lymphoma, BTK activity promotes BCL2 transcription via the nuclear factor κ-light-chain-enhancer of activated B cells (NF-κB) pathway [8]. The blockade of BTK activity attenuates βcR signaling and induces apoptosis by decreasing the levels of antiapoptotic BCL-2, BCL-XL, and Mcl-1 protein [9].

Previous data confirmed that LFM-A13 inhibits phosphoinositide 3-kinase (PI3K) and Epo-induced phosphorylation of erythropoietin receptor (EpoR). This compound was proved to block Janus kinases (JAK2) binding to EpoR, thus breaking the associated intracellular signal transduction pathway [10]. Since a link between BTK and Epo activity exists, we decided to investigate the impact of a combined therapy with Epo and LFM-A13 on apoptosis in colon cancer models [11]. It was demonstrated that Epo-induced signal transduction is inhibited in cells lacking BTK. Moreover, LFM-A13 efficiently inhibits Epo-induced phosphorylation of EpoR and Janus kinase 2 (Jak2), thus breaking the related intracellular signaling pathway [10]. It is well known that Epo used to treat anemia promotes survival, proliferation, and differentiation of the progenitors of erythropoiesis, exerting a proangiogenic and antiapoptotic effect [12,13]. Despite this, the results of our recent study indicate that Epo intensifies the antiproliferative activity of LFM-A13 [14].

In this study, we examined the effect of these compounds on the viability of colon cancer cells, using DLD-1 and HT-29 cell lines. We assessed apoptosis using several biochemical markers, such as phosphatidylserine externalization, loss of mitochondrial membrane potential (MMP), expression of pro-apoptotic protein BAX and of antiapoptotic protein BCL-2. We also explored the effect of the simultaneous use of Epo and LFM-A13 on tumor development in a mouse model of human colorectal cancer. The results demonstrated that the simultaneous use of Epo and LFM-A13 can strongly induce apoptosis in colon cancer cells in vitro as well as reduce tumor volume in colon cancer xenografts in vivo.

## 2. Results

### 2.1. Erythropoietin in Combination with LFM-A13 Decreases Colon Cancer Viability

**Figure 1:**
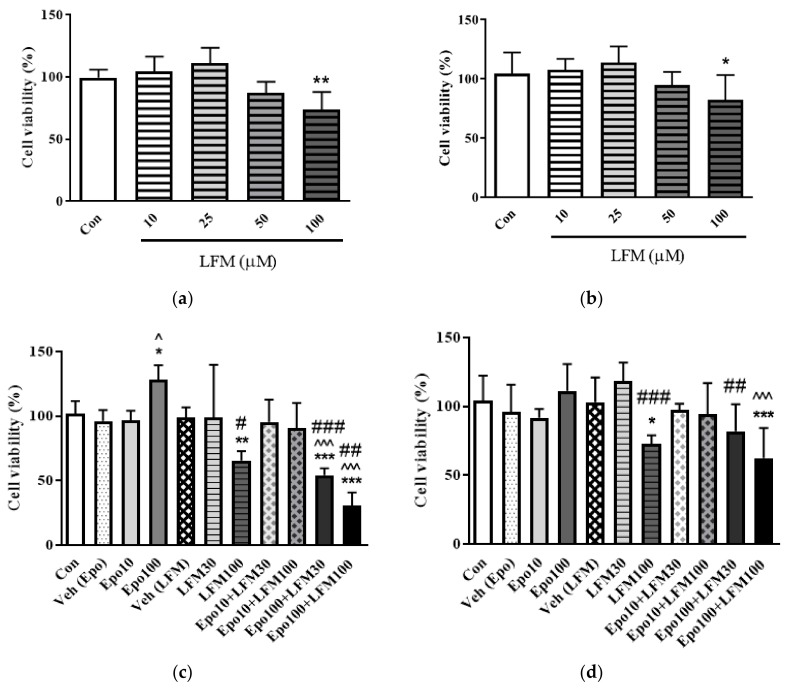

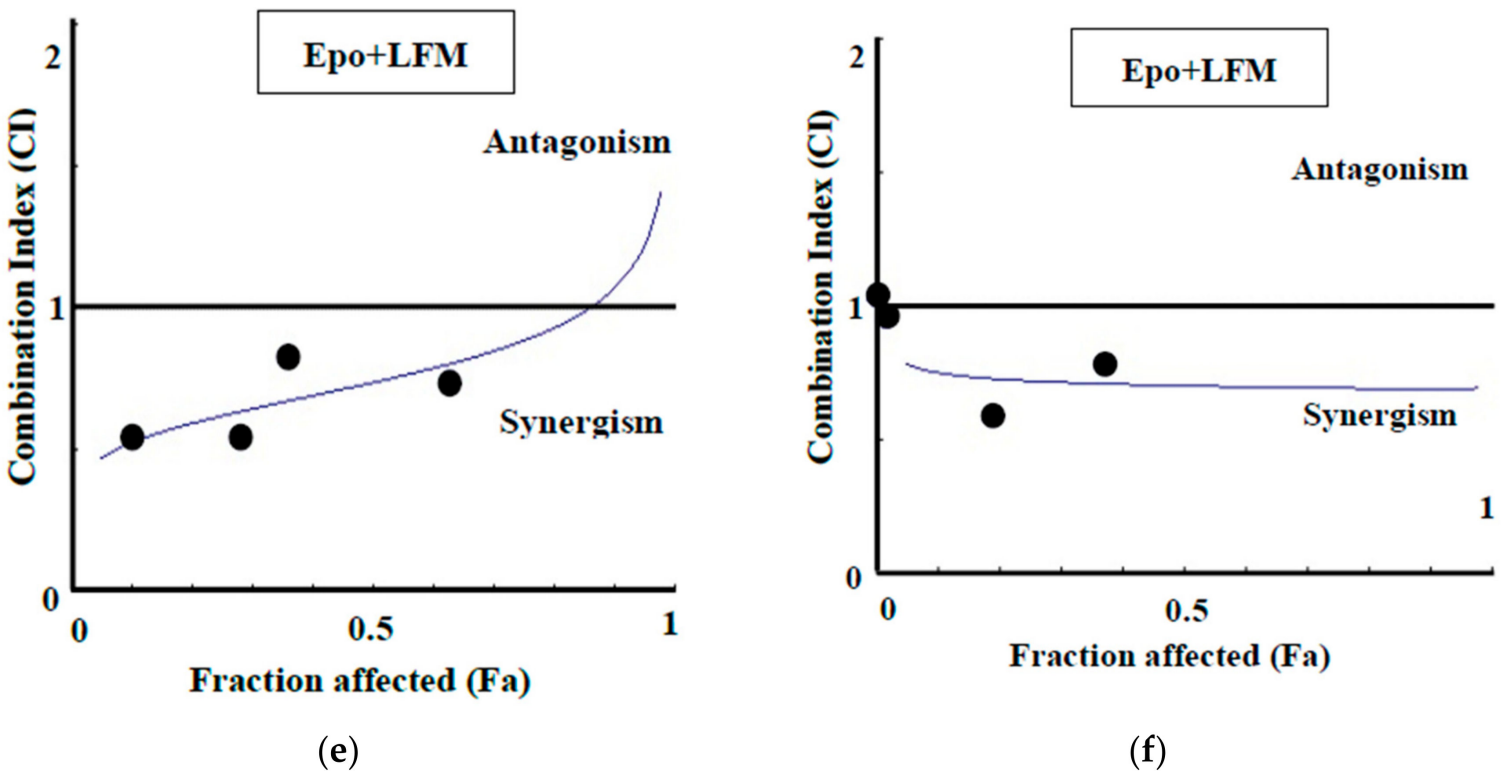
Effects of erythropoietin β (Epo), LFM-A13 (LFM), and their combinations on colon cancer cells. Impact of LFM-A13 (LFM) on cell viability of DLD-1 cells (**a**) and HT-29 cells (**b**); * *p* < 0.05, ** *p* < 0.01 (versus control (Con)). Effect of erythropoietin β (Epo), LFM-A13 (LFM), and their combined activity on cell viability of DLD-1 (**c**) and HT-29 (**d**) colon cancer cells; * *p* < 0.05 (vs. Con), *** *p* < 0.001 (vs. Con); ^ *p* < 0.05 (vs. Epo), ^^^ *p* < 0.001 (vs. Epo); # *p* < 0.05 (vs. LFM-A13), ## *p* < 0.01 (vs. LFM-A13), ### *p* < 0.001 (vs. LFM-A13). Combination index (CI) analysis of erythropoietin (10–100 IU/mL) combined with LFM-A13 (10–100 µM) at a constant ratio in DLD-1 (**e**) and HT-29 (**f**) cells. Synergistic effects are defined as CI < 1, additive effects as CI = 1, and antagonistic effects as CI > 1.

In the first stage of our study, we decided to choose the effective cytotoxic dose of LFM-A13. The MTT test indicated that only the highest dose (100 μM) decreased the viability of colon cancer DLD-1 and HT-29 cells (Figure 1a,b). Therefore, we used this dose in further research. Incubation of DLD-1 cells with 100 IU/mL of Epo and 30 or 100 µM of LFM-A13 decreased cell viability compared with the control (*p* < 0.001, *p* < 0.001 respectively) as well as with cells treated with 100 IU/mL of Epo (*p* < 0.001), 30 µM of LFM-A13 (*p* < 0.001), and 100 µM of LFM-A13 (*p* < 0.01) (Figure 1c). In turn, the reduction in cell viability in the HT-29 line was also statistically significant, but weaker. The combined treatment significantly reduced cell viability only in cells treated with the highest dose (100 µM) of LFM-A13 compared with the control (*p* < 0.001). Adding LFM-A13 (100 µM) to Epo (100 IU/mL) markedly reduced cell viability compared with Epo alone (*p* < 0.001) (Figure 1d). The obtained results indicate that the addition of Epo to LFM-A13 significantly reduced the viability of colon cancer cells compared with LFM-A13 alone. The quantification of the potency of these combinations using the Chou–Talalay method showed synergistic combination indices (CI) for Epo and LFM-A13 in a constant ratio, particularly in the DLD-1 line (Figure 1e). In the case of the HT-29 line, synergism was observed only at the highest doses; at low doses, the calculated CI was close to 1 and thus could represent an additive effect (Figure 1f).

### 2.2. Erythropoietin in Combination with LFM-A13 Induces Apoptosis through Intracellular Signaling Pathway Attenuation 

**Figure 2:**
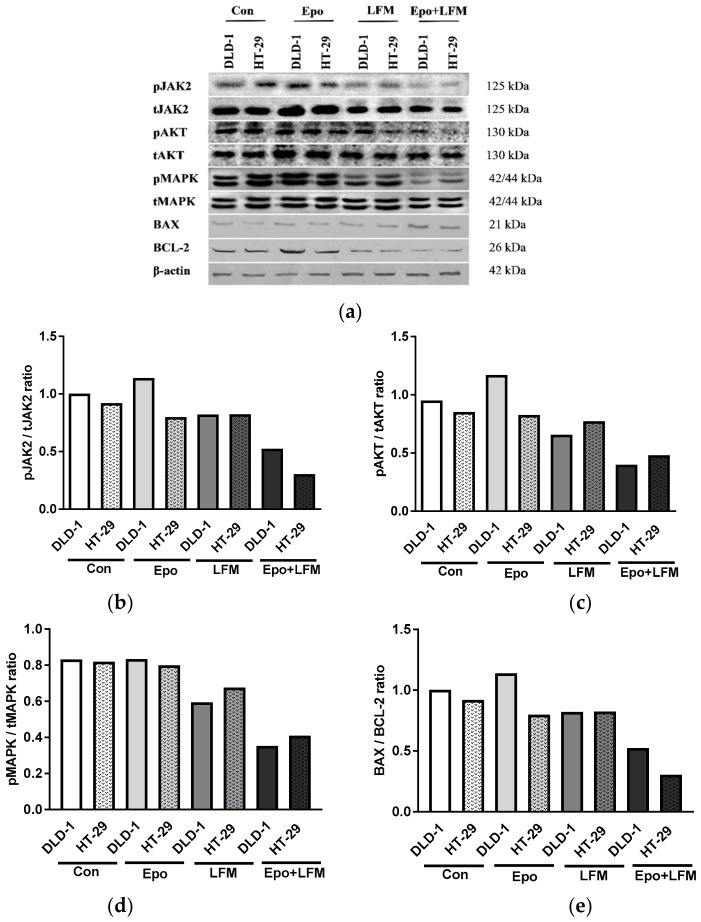
Effect of erythropoietin β (Epo), LFM-A13 (LFM), and their combination on intracellular pathway and apoptosis. Immunoblotting analysis for: (**a**) phospho-JAK2 (pJAK2) and total JAK2 (tJAK2), phospho-AKT (pAKT) and total AKT (tAKT), phospho-MAPK (pMAPK) and total MAPK (tMAPK), BAX and BCL-2 in DLD-1 and HT-29 cells treated with erythropoietin β (Epo 100 IU/mL), LFM-A13 (LFM 100 µM), or their combination for 48 h. The samples used for electrophoresis consisted of 20 µg of protein from six pooled cell extracts (*n* = 6). (**b**–**e**) Band staining was quantified by densitometry; (**b**) pJAK/tJAK, (**c**) pAKT/tAKT, (**d**) pMAPK/tMAPK, (**e**) BAX/BCL-2 ratio the band intensities of the phospho-proteins are normalized with respect to the intensities of the respective total protein bands.

To illustrate the mechanism underlying the regulation of intracellular signal by the Epo and LFM-A13 combination, we investigated the status of the JAK2, AKT, and mitogen-activated protein kinases (MAPK) pathways in colon cancer cells. As shown in Figure 2, simultaneous use of Epo and LFM-A13 failed to induce efficient phosphorylation of JAK2, AKT, and MAPK. Moreover, LFM-A13 with Epo further inhibited the intracellular signaling pathway in comparison with LFM-A13 alone in both DLD-1 and HT-29 cells.

A 48 h treatment with Epo and LFM-A13 contributed to a marked increase in the expression of BAX compared with control cells in both lines. The combination treatment intensified BAX expression more than LFM-A13 alone. Simultaneously, we found a decrease in the expression of BCL-2 after incubation with Epo and LFM-A13 compared with the control cells. Incubation with Epo led to an increase in BCL-2 expression particularly in DLD-1 cells, which could be due to the presence of EpoR receptors in these cells. An increase in the BAX/BCL-2 ratio correlated with an increase in the apoptotic process. In our study, the ratio of BAX to BCL-2 shifted in favor of BAX compared with the control, which confirms the intensification of this process (Figure 2).

**Figure 3:**
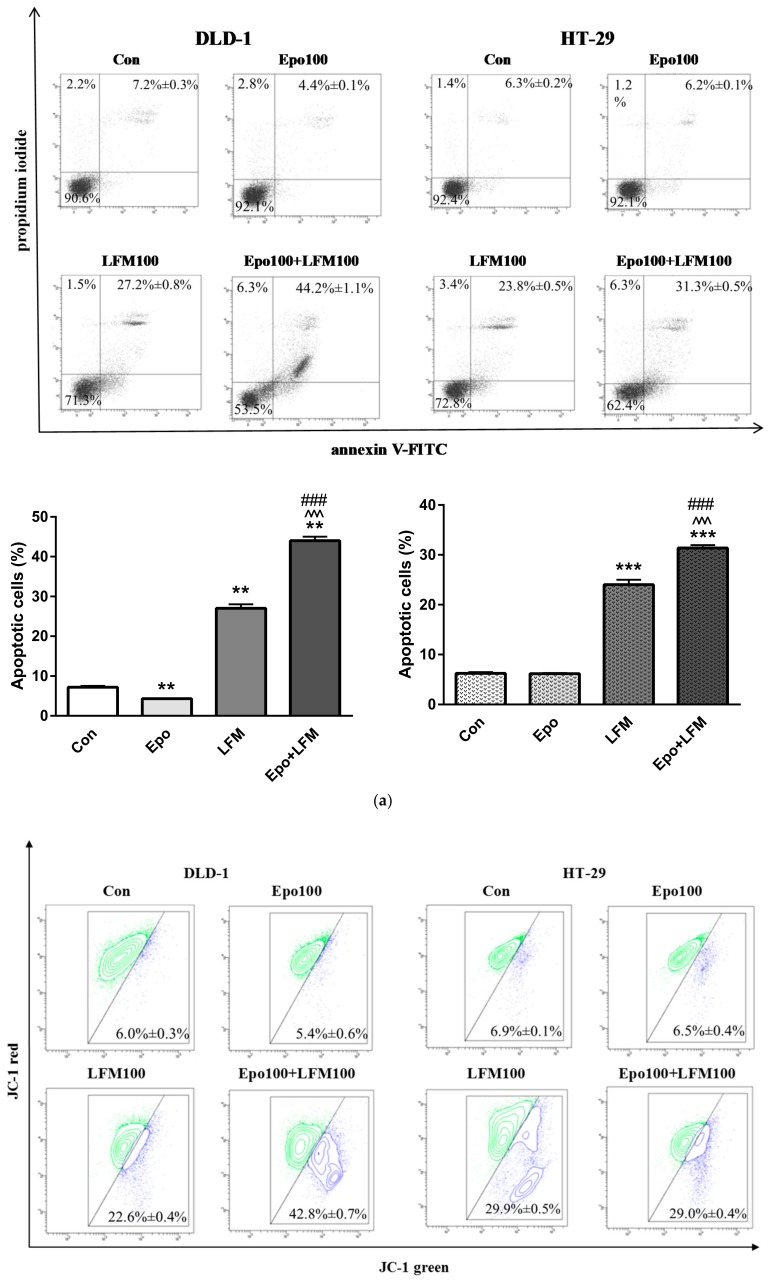

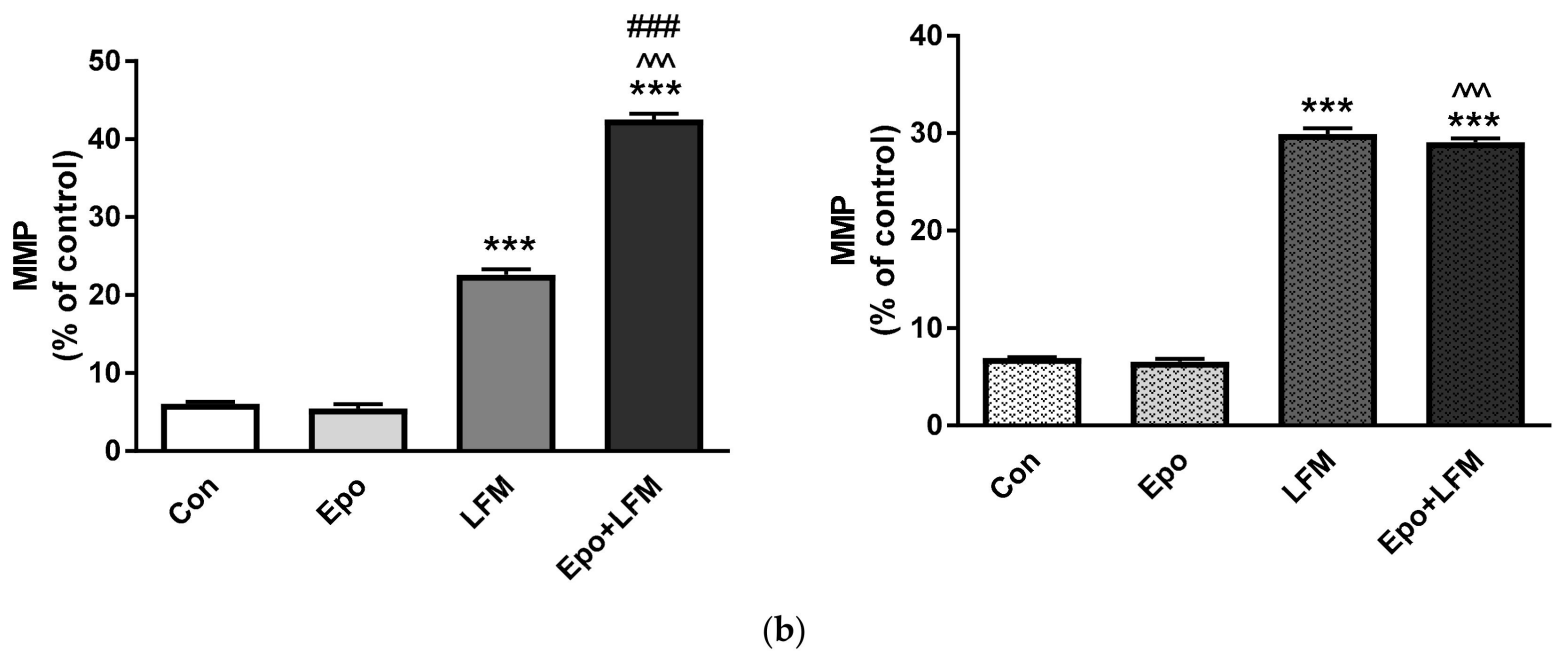
Effect of erythropoietin β (Epo), LFM-A13 (LFM), and their combinations on intracellular pathway and apoptosis. (**a**) Representative flow cytometry dot plots for Annexin V–FITC (fluorescein isothiocyanate) assay of DLD-1 and HT-29 cells incubated with Epo (Epo100, 100 IU/mL) and LFM-A13 (LFM100, 100 μM) for 48 h (mean ± SD; *n* = 3). The live cells appear at the lower left corner in the plots; the early apoptotic cells appear at the lower right corner; the necrotic cells appear at the upper left corner; the dead cells appear at the upper right corner. Left panel: The percentage of apoptotic DLD-1 cells incubated with Epo and LFM-A13 is shown in the bar diagram as mean ± SD (*n* = 3). Right panel: The percentage of apoptotic HT-29 cells incubated with Epo and LFM-A13 is shown in the bar diagram as mean ± SD (*n* = 3); * *p* < 0.05 (vs. Con), ** *p* < 0.01 (vs. Con), *** *p* < 0.001 (vs. Con); ^ *p* < 0.05 (vs. Epo), ^^^ *p* < 0.001 (vs. Epo); # *p* < 0.05 (vs. LFM-A13), ### *p* < 0.001 (vs. LFM-A13). (**b**) Representative dot plots presenting the loss of mitochondrial membrane potential (MMP) in DLD-1 and HT-29 cells incubated with Epo (Epo100, 100 IU/mL) and LFM-A13 (LFM100, 100 μM) for 48 h (mean ± SD; *n* = 3). Cells with normal MMP are shown on the right side of the plots, cells with decreased MMP on the left side of the plots. The graphs show the percent of cells with decreased mitochondrial membrane potential in DLD-1 (left panel) and HT-29 cells (right panel).

Apoptosis was detected by staining with annexin V, a phospholipid-binding protein commonly used for the detection of early apoptosis. For assessment of the cell death mode induced by Epo+LFM-A13, flow cytometric analysis was carried out using annexin V and propidium iodide (PI). Dual-labeling for annexin V and PI allows the distinction between live cells (annexin-V−/PI−), early apoptotic cells (annexin-V+/PI−), late apoptotic cells (annexin-V+/PI+), and necrotic cells (annexin-V−/PI+) (Figure 3a). As shown in Figure 3a, after 48 h of treatment with Epo+LFM-A13, we observed a significant accumulation of annexin V-positive cells in both DLD-1 and HT-29 cells. In-term administration of Epo slightly inhibited apoptosis in both tested lines after 48 h incubation compared with the control. We found that the apoptotic effect of Epo+LFM-A13 was stronger than that evoked by LFM-A13 alone, which resulted in fullest induction of apoptosis in both DLD-1 and HT-29 cancer cell lines.

To examine the cellular mechanism underlying Epo+LFM-A13-induced intrinsic apoptosis in DLD-1 and HT-29 cells, we evaluated changes in the mitochondrial transmembrane potential using flow cytometry analysis. We observed that Epo+LFM-A13 increased the number of cells with decreased levels of MMP in both DLD-1 and HT-29 cells at 24 h (Figure 3b). Mitochondria a play key role in the propagation of apoptosis [15,16]. It is known that, at an early stage, apoptotic stimuli alter the mitochondrial transmembrane potential, which can be measured by determining the fluorescence of the dye JC-1. As shown in Figure 3b, Epo+LFM-A13 in DLD-1 and HT-29 cells induced an increase in the proportion of cells with depolarized mitochondria. We observed that the effect of Epo+LFM-A13 was stronger than that caused by LFM-A13 alone. These results are consistent with those obtained in the annexin V and PI test.

### 2.3. Anti-Colon Cancer Activity of the Simultaneous Use of Epo and LFM-A13 in Mouse Xenografts

**Figure 4:**
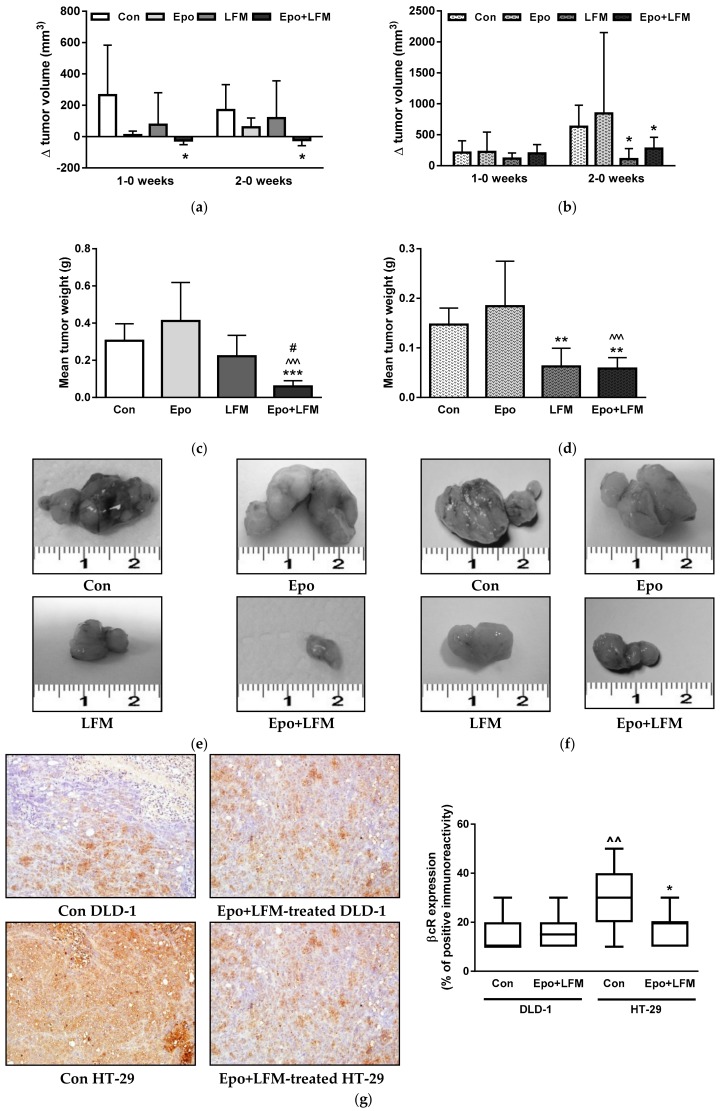
Effect of the combined activity of LFM-A13 and erythropoietin β on a mouse model of colorectal cancer. Impact on tumor volume in DLD-1 (**a**) and HT-29 (**b**) xenografts; 0—before substance administration, 1—after first week of substance administration, 2—after second week of substance administration. The results are presented as mean values ± SD, *n* = 5–10. Effect of the combined activity of LFM-A13 (10 mg/kg b.m.) and erythropoietin β (Epo, 600 IU/kg b.m.) on tumor weight in DLD-1 (**c**) and HT-29 (**d**) xenografts. The results are presented as mean values ± SD, *n* = 10. * *p* < 0.05 (vs. Con), ** *p* < 0.01 (vs. Con), *** *p* < 0.001 (vs. Con); ^ *p* < 0.05 (vs. Epo), ^^ *p* < 0.01 (vs. Epo), ^^^ *p* < 0.001 (vs. Epo); # *p* < 0.05 (vs. LFM-A13). Representative weights of the tumors dissected from the nude mice untreated or treated with Epo, LFM-A13, and Epo+LFM-A13 in DLD-1 (**e**) and in HT-29 (**f**). (**g**) Positive expression of βcR in the cytoplasm of colon cancer xenografts. Left panel: expression in control and Epo-LFM-A13-treated DLD-1 and HT-29 xenografts (H&E staining; magnification, ×200). Right panel: a box-and-whisker plot of percentage βcR expression in DLD-1 and HT-29 tumor xenografts. The results are presented as medians (minimum–maximum), *n* = 10, * *p* < 0.05 (vs. Con), ^ *p* < 0.05 (Con in DLD-1 vs. Con in HT-29 xenografts).

In order to confirm the results of the in vitro experiments, we performed animal studies. In DLD-1 xenografts receiving Epo+LFM-A13, differences in tumor growth between the first and the zero week as well as the second and the zero week were significantly smaller compared with the control (*p* < 0.05, *p* < 0.05, respectively, Figure 4a). In HT-29 xenografts treated with Epo+LFM-A13, the growth increment was significantly smaller only between the second and the zero week compared with the control group (*p* < 0.05, Figure 4b). Additionally, in HT-29 xenografts, a smaller growth increment was obtained after LFM-A13 therapy in the same period compared with the control (*p* < 0.05). As shown in Figure 4c,d, the tumor weights of DLD-1 and HT-29 xenografts treated with a combination of Epo+LFM-A13 were significantly lower compared with the control (*p* < 0.001 and *p* < 0.01, respectively). In HT-29 xenografts, LFM-A13 significantly decreased tumor development compared with the control animals (*p* < 0.01). The tumors of mice treated with Epo+LFM-A13 were five times smaller compared with the control DLD-1 xenografts, and two-and-a-half times smaller compared with the control HT-29 xenografts (Figure 4e,f). Cytoplasmic anti-βcR (β common receptor) staining prevailed in all positive slides. In the present study, weak positive immunoreactivity for βcR was found in nine control DLD-1 xenografts (90% of all cases); one (10% of all cases) exhibited medium staining. We obtained similar results in Epo+LFM-13-treated animals. Interestingly, in control HT-29 xenografts, medium-positive immunoreactivity for βcR was observed in seven animals (70% of all cases); however, one animal (10% of all cases) exhibited strong staining and two (20% of all cases) medium staining. In Epo+LFM-A13-treated xenografts, weak immunoreactivity was found in nine cases (95% of positive tumors), while medium immunoreactivity was detected in one case (10% of positive tumors) (Figure 4g). These results indicate that the combined therapy has potent anticancer activity, in which βcR receptors are probably involved.

## 3. Discussion

Despite the use of combination therapies in many patients with colon cancer, fully satisfactory results have not been achieved. The current work investigates the effect of the simultaneous use of erythropoietin (Epo) and LFM-A13 (BTK inhibitor) on the viability of DLD-1 and HT-29 colon cancer cells and on their growth in xenografts, as well as its putative mechanism of cytotoxicity. The novel drug combination of Epo and LFM-A13 that we used proved to exert a high cytotoxic effect in DLD-1 and HT-29 colon cancer cells. The simultaneous use of these agents induced apoptosis in the treated cells. We extended these observations in vivo by demonstrating that, in DLD-1 xenografts, LFM-A13 alone was able to delay tumor development, while a combination of Epo with LFM-A13 led to significant tumor regression.

The evaluation of the cytotoxicity of Epo and LFM-A13 used together in both DLD-1 and HT-29 colon cancer cells demonstrated that this combination had a greater antiproliferative effect than the use of the latter drug alone. During the last decade, a plethora of studies has suggested that LFM-A13 exhibits antiproliferative activity. LFM-A13 revealed mitotic arrest and prevented bipolar spindle assembly formation in human cancer cell lines and glioblastoma cells [17]. Uckun et al. also confirmed the antiproliferative activity profile of LFM-A13 and reported that this agent blocks cell division in a zebra fish embryo model at the 16-cell stage of embryonic development [18]. It has also been shown that the inhibition of BTK expression by LFM-A13 decreased the proliferation of prostate cancer cells, but not of normal prostate epithelial cells, which express very little BTK [19]. On the basis of these results and the report indicating a relationship between BTK, LFM-A13, and erythropoietin [10], we used a combination of these agents. The results of our study indicate that the addition of Epo to LFM-A13 enhances the antiproliferative activity of LFM-A13. Similar results were obtained in previous studies, in which we observed that the combined use of Epo and cytotoxic agents such as 5-fluorouacil and active metabolite of irinotecan (SN-38) enhanced the antitumor effect on DLD-1 human colon cancer cells [20].

Then, we attempted to clarify the potential mechanism of action. On one hand, the activity of BTK, which is involved in the activation of the signaling pathways responsible for maturation, viability, and cell differentiation, is inhibited by LFM-A13. In turn, we supposed that Epo may increase pBTK expression and enhance the antiproliferative action of LFM-A13 through the elevation of the levels of its target proteins. Our experiments, carried out by flow cytometry assessment of annexin V binding, revealed that LFM-A13 increased the number of DLD-1 and HT-29 apoptotic cells. Epo with LFM-A13 further enhanced these effects and induced a high level of apoptosis in both DLD-1 and HT-29 (Figure 2).

Mitochondria play a key role in the apoptosis of mammalian cells and undergo characteristic functional and structural changes at an early stage of the death process [21]. Almost all apoptotic pathways concentrate on the mitochondria, such as the decrease of the mitochondrial transmembrane potential. A decrease of MMP characterizes an early and already invariant stage of apoptosis [15,22]. Incubation with Epo and LFM-A13 led to a stronger decrease of the mitochondrial membrane potential in DLD-1 than incubation with LFM-A13 alone.

We also examined the mechanism through which the used combination of compounds was able to induce apoptosis. By profiling the relevant pathways, we found that simultaneous use of Epo and LFM-A13 resulted in the activation of pro-apoptotic BAX and downregulation of antiapoptotic BCL-2, which may cause a release of cytochrome C from the mitochondria in DLD-1 and HT-29 cells. It was previously shown that a high ratio of BAX to BCL-2 can lead to loss of mitochondrial membrane potential, causing a release of cytochrome c and consequently inducing apoptosis [23,24]. It was also demonstrated that another BTK inhibitor—ibrutinib—induced concentration-dependent apoptosis in both Mino or Jeko-1 cells and decreased the levels of antiapoptotic BCL-2, BCL-XL, and Mcl-1 proteins [9]. Moreover, simultaneous use of ibrutinib and bortezomib synergistically increased mitochondrial injury and apoptosis in diffuse large B cell lymphoma (D LBCL) and Mantle cell lymphoma (MCL) cells, with significant AKT and NF-κB inactivation, decrease in Mcl-1, BCL-XL, and X-linked inhibitor of apoptosis protein (XIAP), and increased DNA damage and endoplasmic reticulum stress [25]. Herman et al. demonstrated dose- and time-dependent cytotoxicity of ibrutinib in chronic lymphocytic leukemia (CLL) via an apoptotic pathway dependent on caspase-3 [26].

The results of this study and other data clearly indicate that Epo increases BCL-2 expression in numerous cancer cells [27,28]. According to the literature data, increased levels of the antiapoptotic BCL-2 protein have been associated with resistance to cytotoxic drugs and advanced stages of multiple myeloma [29]. In the current study, we found that the administration of Epo with LFM-A13 resulted in increased apoptosis in both colon cancer lines, as evidenced by the clear increase in the BAX/BCL-2 ratio. Interestingly, our previous data also indicated that the combination of Epo with the anticancer agent H_2_O_2_ led to decreased BCL-2 and increased BAX expression [30].

Other putative mechanisms of apoptosis enhancement may be associated with the regulation of BTK activity. BTK can mediate downstream signaling to regulate cell growth, differentiation, and apoptosis [31]. This kinase is an upstream activator of multiple antiapoptotic signaling molecules and networks, such as signal transducer and activator of transcription 5, NF-κB, and AKT. It is well known that BTK plays a crucial role in the B cell tumors development, by activating antiapoptotic pathways. Phosphorylated BTK activates phospholipase C2, leading to downstream activation of protein kinases and ultimately to the activation of transcription factor NF-κB. The inhibition of apoptosis occurs as a result of the stimulation of the NF-κB pathway. This cascade of events has been linked with the proliferation and survival of B cell malignancies [25]. BTK also inhibits Fas-ligand-mediated apoptosis. Loss of BTK caused B cells to be more sensitive to Fas-induced apoptosis in response to certain death signals [4,31,32].

Moreover, inhibiting BTK increases the expression of apoptosis-related genes [7]. Downregulation of this kinase with iRNA or inhibition with pharmacological inhibitors leads to apoptosis, whereas its overexpression inhibits apoptosis induced by doxorubicin in breast cancer cells [33]. B lymphocytes and myeloid cells with low BTK activity tend to undergo apoptosis and exhibit decreased proliferation [31]. Findings from the literature data showed that BTK is aberrantly expressed in DLD-1 and HT-29 colon carcinoma cells and that LFM-A13 was able to decrease the phosphorylation of this kinase [3,34]. We speculate that the stronger downregulation of BTK after simultaneous use of Epo and LFM-A13 may be the cause of the enhanced proapoptotic effects. This indicates that the decrease in proliferation in DLD-1 and HT-29 cells may be due to, at least in part, increased apoptosis. This is in line with the data of Guo et al., who showed that inhibition of BTK contributes to the induction of apoptosis in prostate cancer cells [19]. Inhibition of this kinase using LFM-A13 also resulted in increased apoptosis in BT474 and MCF-7 breast cancer cells [7]. The potential proapoptotic effect of a combined therapy with Epo and LFM-A13 could also be related to inhibition of p53 protein phosphorylation due to the blocking of BTK activity. Althubiti et al. observed that BTK expression increased the stability of p53 and its transactivation abilities [35,36]. Tumor protein p53 is a nuclear transcription factor that controls the expression of many of the genes responsible for apoptosis (BAX, BAK). BTK has an impact on p53 phosphorylation, which suggests that it is a crucial upstream regulator of the p53 pathway. Consequently, the cellular responses to p53 upregulation, such as apoptosis, were greatly influenced by the expression of BTK [35,36]. Further studies are needed to clarify the mechanism, because it is not fully understood.

Our in vitro study demonstrated the beneficial effect of LFM-A13 in combination with erythropoietin on antitumor activity in both tested colon cancer cell lines. We investigated whether erythropoietin can enhance in vivo colon cancer cell growth and examined the pharmacodynamics features of the promising combination in DLD-1 and HT-29 xenografts. In this model, 100% of mice survived for 3 weeks after inoculation with 1 × 10^6^ DLD-1 and HT-29. LFM-A13 in a nontoxic low dose of 10 mg/kg decreased HT-29 and DLD-1 xenograft tumor growth. Our results confirm the results of a previous study by Uckun et al., who reported a delay in tumor progression in the MMTV/Neu transgenic mouse model of HER2-positive breast cancer after LFM-A13 administration [18]. Moreover, we found a greater reduction in tumor volume in the Epo-and-LFM-A13-treated group compared with the control and the group treated with LFM-A13 alone. We also observed that DLD-1 xenografts were more responsive to this therapy compared with HT-29 xenografts. In the case of HT-29, not all results were consistent under both in vitro and in vivo conditions (Figure 2). It is difficult to explain this unequivocally; however, the observed effects were not opposite, and the trend remained the same. It is well known that erythropoietin receptors (EpoR) are located not only in erythroid cells, but also in the pituitary glands and in breast, brain, colon, kidney, lung, ovarian, endometrial, prostate, and lymphoma cancer cells [37,38]. Also, β common receptor (βcR), a shared receptor subunit of interleukin-3 (IL-3), IL-5, and granulocyte–macrophage colony stimulation factor (GM-CSF) receptors, mediates the non-hematopoietic activity of Epo in various cell types [39]. In non-hematopoietic tissue, Epo may act via the epinephrine B4 receptor (EphB4). It has been proven that Epo binds to the EpoR–EphB4 heterodimer as well as to the EpoR–βcR–EpoR heterotrimer [37]. The observed reduction in tumor volume in HT-29 xenografts after treatment with Epo–LFM–A13 appears to be dependent on βcR. High expression of this receptor was observed in control HT-29 xenografts; however, its expression was significantly reduced in the group of treated animals. Thus, lowering the expression of the receptor, which has been shown to be essential to the Epo-mediated extra-hematopoietic tissue protective effects, may lead to abolished Epo-induced NO production via the Src/JAK2/Akt signaling pathway and to reduced vascular endothelial growth factor (VEGF) production and angiogenic potency [40].

## 4. Materials and Methods 

### 4.1. Reagents

RPMI-1640, McCoy’s 5a medium, fetal bovine serum, penicillin, and streptomycin were obtained from ATCC (American Type Culture Collection, Manassas, VA, USA). Erythropoietin β (NeoRecormon, Roche, Basel, Switzerland) was purchased from Roche. LFM-A13 (2-cyano-*N*-(2,5-dibromophenyl)-3-hydroxy-2-butenamide) was a Tocris product (Bristol, UK). Stock solutions of LFM-A13 were prepared in methanol and stored at −20 °C. Because of the poor aqueous solubility of LFM-A13, the compound was dissolved in 0.01% dimethyl sulfoxide (DMSO) in phosphate-buffered saline (PBS), according to the manufacturer’s instructions. A similar quantity of DMSO was added to the control preparations.

### 4.2. Cell Cultures

The first stage of research involved in vitro experiments using two lines of colon cancer cells, DLD-1 (ATCC, Manassas, VA, USA, Cat# CCL-221, RRID:CVCL_0248) and HT-29 (ATCC, Cat# HTB-38, RRID:CVCL_0320). Both lines are considered adhesive and quick-growing. DLD-1 cells contain the *EpoR* gene and protein and histologically are the most similar to primary tumor cells, while the cell line HT-29 is a negative control for the *EpoR* gene. The cell line DLD-1 was isolated from the epithelial tissue of a colon cancer adenocarcinoma (stage C according to Dukes classification) of an adult man. It exhibits the presence of the oncogenes myc, myb, ras, fos, sis, and p53 and the absence of the oncogenes abl, ros, and src. The cell line HT-29 was isolated from a colon adenocarcinoma of a 44-year-old woman. In in vitro cultures, it exhibits a morphology typical of epithelial cells, but it does not differentiate into cells making a brush border. A huge part of the HT-29 cell population is composed of goblet cells, so this line produces great amounts of intestinal mucus. HT-29 cells exhibit the presence of the oncogenes myc, myb, ras, myb, fos, sis, and p53 and the absence of the oncogenes abl, ros, and src.

### 4.3. Exogenous Erythropoietin and LFM-A13 Administration 

DLD-1 and HT-29 colon cancer cells were incubated for 48 h with exogenous erythropoietin β at a concentration of 100 IU/mL, LFM-A13 at a concentration of 100 µM, and a combination of these drugs (Epo+LFM-A13). Epo [41] and LFM-A13 [42,43] concentrations were selected on the basis of data from the literature. In these types of experiments, Epo at a concentration of 100 IU/mL has been commonly used by other researchers [44,45].

### 4.4. Cell Viability Assay

The assay was carried out in accordance with the method described by Mosmann using 3-(4,5-dimethylthiazol-2-yl)-2,5-diphenyltetrazolium bromide (MTT) [46]. Confluent cells cultured for 48 h with various concentrations of the tested compounds in 6-well culture plates were washed three times with PBS and next incubated at 37 °C in a 5% CO_2_ incubator for 4 h in 1 mL of MTT solution (0.5 mg/mL of PBS). The medium was discarded, and 1 mL of 0.1 M HCl in absolute isopropanol was added to colon cancer cells. The absorbance of the transformed dye in living cells was measured at 570 nm. The viability of DLD-1 and HT-29 cells cultured in the presence of Epo and LFM-A13 was calculated as a percentage of the control cells. The experiments were carried out three times.

### 4.5. Western Blotting

Western blotting was performed using a standard method described previously [12]. The nitrocellulose membranes were incubated with following antibodies: mouse anti-BAX (Sigma-Aldrich, St. Louis, MO, USA, Cat# WH0000581M1, RRID:AB_1840183,), mouse anti-BCL-2 (Sigma-Aldrich Cat# B3170, RRID:AB_258541), rabbit anti-phospho Akt1/2/3 (Santa Cruz Biotechnology, Dallas, TX, USA, Cat# sc-7985 also sc-7985-R, RRID:AB_667741), rabbit anti-Akt1/2/3 (Santa Cruz Biotechnology, Cat# sc-8312, RRID:AB_671714), rabbit anti-phospho-MAPK (Thermo Fisher Scientific, Waltham, MA, USA, Cat# PA1-14302, RRID:AB_1086514), rabbit anti-MAPK (Thermo Fisher Scientific Cat# PA5-14425, RRID:AB_2141578), rabbit anti-pospho JAK2 (Cell Signaling Technology, Leiden, The Netherlands, Cat# 3771S, RRID:AB_330403), rabbit anti-JAK2 (Cell Signaling Technology Cat# 4040S, RRID:AB_10691469), and mouse anti- β-Actin (Sigma-Aldrich Cat# A5316, RRID:AB_476743).

### 4.6. Flow Cytometry Assessment of Annexin V Binding

The apoptosis Detection Kit II (BD Pharmingen, San Jose, CA, USA) was used to characterize the mode of cell death induced by Epo plus LFM-A13. Colon cancer cells (10,000 cells measured) were examined in a flow cytometer (BD FACSCanto II flow cytometer, San Jose, CA, USA). Annexin V has high affinity for phosphatidylserine and therefore can be used to recognize cells in all stages of programed cell death. Propidium iodide (PI) is used as a DNA stain in cells exposing a disrupted cell membrane and were used to evaluate the late apoptotic and dead cells. The optimal parameter settings were discovered using a positive control (cells incubated with 3% formaldehyde buffer for 30 min on ice). FACSDiva software (BD Biosciences Systems, San Jose, CA, USA) was used for the analysis of the results.

### 4.7. Analysis of Mitochondrial Membrane Potential

The disturbances of the mitochondrial membrane potential were evaluated using the lipophilic cationic probe 5,5′,6,6′-tetrachloro-1,1′,3,3′-tetraethylbenzimidazolcarbocyanine iodide (JC-1 MitoScreen kit, BD Biosciences). Unfixed colon cancer cells were washed twice in PBS and resuspended in PBS supplemented with 10 μg/mL JC-1. Then, the cells were incubated at room temperature in the dark for 15 min, washed, and resuspended in PBS for immediate BD FACSCanto II flow cytometry analysis. The FACSDiva software (BD Biosciences Systems) was used to calculate the percentage of cells with disrupted MMP.

### 4.8. Establishment of Xenograft 

**Figure 5:**
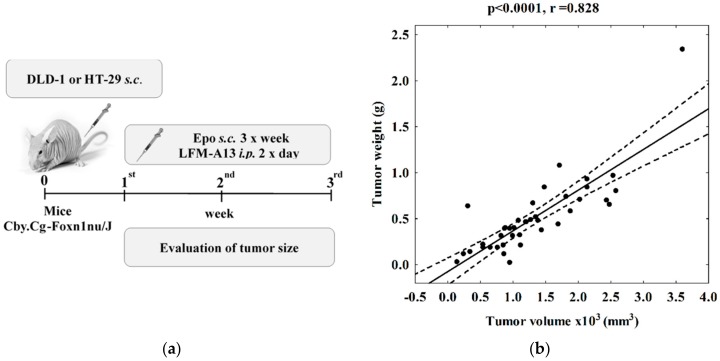
Human tumor xenografts in a nude mouse model. (**a**) Experimental plan. (**b**) Positive correlation between tumor volume and weight at mouse sacrifice (*p* < 0.0001, *r* = 0.828). Solid line—regression; dashed lines—95% confidence intervals for regression line.

Animal experiments were approved by the Local Ethics Committee (date of approval: 24 April 2013; identification code: 31/2013). Animal studies are reported in compliance with the ARRIVE guidelines [47]. Xenograft animal models were obtained as described previously [14] using DLD-1 and HT-29 cells (50 μL suspension containing 1 × 10^8^ cells for each animal, Figure 5a). In previous studies, we showed no effect of the Epo solvent on the number of colon cancer cells [20] and, in accordance with the 3R, we reduced the number of animals [48]. In order to evaluate the validity of the tumor volume measurements, we correlated the tumor weight at mouse sacrifice with the tumor size. A subsequent analysis confirmed the presence of a positive correlation (*r* = 0.828, *p* < 0.0001) between these parameters and thus confirmed the validity of this method of measurement (Figure 5b).

### 4.9. Immunocytochemistry

Formalin-fixed, paraffin-embedded tissue slides were cut on the microtome into sections with a thickness of 4 mm and processed as described previously [49]. The antibody used (βcR (7C6); Cat# sc-103, RRID:AB_626741) was obtained from Santa Cruz Biotechnology.

### 4.10. Statistical Analysis 

The obtained results were statistically analyzed. The Shapiro–Wilk test was used to assess characteristics consistent with normal distribution, the Student’s *t*-test was used for comparisons between two groups, and the Mann–Whitney test was used for features inconsistent with the distribution. For comparisons of more than two groups, the analysis of variance with Bonferroni post hoc test or the Kruskal–Wallis test were used. Student’s *t*-test for pairs and Wilcoxon signed-rank test were used to analyze the measurements in groups at time intervals. In order to evaluate the correlations between the studied parameters, the Pearson correlation coefficient was used. In all experiments, the mean values for 4–10 assays ±S.D. were calculated using Statistica 10 software (Statistica 10, RRID:SCR_015627). A level of *p* < 0.05 was considered statistically significant. The calculations were performed using GraphPad 7.02 Prism software (GraphPad Software, Inc., La Jolla, CA, USA; Available online: https://www.graphpad.com/scientific-software/prism, RRID:SCR_015807). CI values were determined by CompuSyn software (ComboSyn, Inc., Paramus, NJ, USA; Available online: http://www.combosyn.com) [50]. The potency of the combination was evaluated using the Chou-Talalay method, which calculates a combination index (CI) that is interpreted as follows: CI < 1, synergistic effect; CI = 1, additive effect; CI > 1, antagonistic effect. The bands were quantified using Image J 1.50a software (Available online: https://imagej.nih.gov, RRID:SCR_003070).

## 5. Conclusions

Collectively, we discovered that adding erythropoietin to LFM-A13 significantly intensified the proapoptotic activity of LFM-A13. The combined treatment exhibited antiproliferative activity against colon cancer cells both in in vitro and in vivo via an increasing apoptosis. The outcomes of our study suggest that the proposed combined therapy may be useful as a new approach employing chemosensitizing and apoptosis-promoting anticancer agents for the treatment of colon cancer patients. However, further preclinical and clinical studies are needed to confirm our findings.

## 6. Patents

The results were submitted in the patent application No. P.413921.

## Abbreviations

AKT	protein kinase B
BAX	apoptotic protein
BCL-2	antiapoptotic protein
βcR β	β common receptor
BTK	Bruton’s tyrosine kinase
BT474	cell line of human ductal carcinoma
DLD-1	cell line of human colorectal adenocarcinoma
EphB4	epinephrine B4 receptor
Epo	erythropoietin
EpoR	erythropoietin receptor
FAS	Fas-associated protein with death domain
FADD	FLICE inhibitory proteins: regulators of death receptor-mediated apoptosis
GM-CSF	granulocyte–macrophage colony stimulation factor
HER2	human epidermal growth factor 2
HT-29	cell line of human colorectal adenocarcinoma
IL	interleukin
JAK2	Janus kinase 2
LFM-A13	Bruton’s tyrosine kinase inhibitor
MAPK	mitogen-activated protein kinases
MCF-7	cell line of human breast cancer
MMP	mitochondrial membrane potential
MYC	a regulator gene that codes for a transcription factor
NF-κB	nuclear factor κ-light-chain-enhancer of activated B cells
p-Akt	phosphorylated protein kinase B
PH	pleckstrin homology
PI3K	phosphoinositide 3-kinase
VEGF	vascular endothelial growth factor
